# Chemical priming enhances plant tolerance to salt stress

**DOI:** 10.3389/fpls.2022.946922

**Published:** 2022-09-07

**Authors:** Faisal Zulfiqar, Muhammad Nafees, Jianjun Chen, Anastasios Darras, Antonio Ferrante, John T. Hancock, Muhammad Ashraf, Abbu Zaid, Nadeem Latif, Francisco J. Corpas, Muhammad Ahsan Altaf, Kadambot H. M. Siddique

**Affiliations:** ^1^Department of Horticultural Sciences, Faculty of Agriculture and Environment, The Islamia University of Bahawalpur, Bahawalpur, Pakistan; ^2^Mid-Florida Research and Education Center, Environmental Horticulture Department, Institute of Food and Agricultural Sciences, University of Florida, Apopka, FL, United States; ^3^Department of Agriculture, University of the Peloponnese, Kalamata, Greece; ^4^Department of Food, Environmental and Nutritional Science, Università degli Studi di Milano, Milano, Italy; ^5^Department of Applied Sciences, University of the West of England, Bristol, United Kingdom; ^6^Institute of Molecular Biology and Biotechnology, The University of Lahore, Lahore, Pakistan; ^7^Plant Physiology and Biochemistry Section, Department of Botany, Aligarh Muslim University, Aligarh, India; ^8^Antioxidant, Free Radical and Nitric Oxide in Biotechnology, Food and Agriculture Group, Department of Biochemistry, Cell and Molecular Biology of Plants, Estación Experimental del Zaidín, Consejo Superior de Investigaciones Científicas (CSIC), Granada, Spain; ^9^School of Horticulture, Hainan University, Haikou, China; ^10^The UWA Institute of Agriculture, The University of Western Australia, Perth, WA, Australia

**Keywords:** antioxidants, bioregulator, hydrogen peroxide, hydrogen sulfide, nitric oxide, chitosan, molecular hydrogen, thiamine

## Abstract

Salt stress severely limits the productivity of crop plants worldwide and its detrimental effects are aggravated by climate change. Due to a significant world population growth, agriculture has expanded to marginal and salinized regions, which usually render low crop yield. In this context, finding methods and strategies to improve plant tolerance against salt stress is of utmost importance to fulfill food security challenges under the scenario of the ever-increasing human population. Plant priming, at different stages of plant development, such as seed or seedling, has gained significant attention for its marked implication in crop salt-stress management. It is a promising field relying on the applications of specific chemical agents which could effectively improve plant salt-stress tolerance. Currently, a variety of chemicals, both inorganic and organic, which can efficiently promote plant growth and crop yield are available in the market. This review summarizes our current knowledge of the promising roles of diverse molecules/compounds, such as hydrogen sulfide (H_2_S), molecular hydrogen, nitric oxide (NO), hydrogen peroxide (H_2_O_2_), melatonin, chitosan, silicon, ascorbic acid (AsA), tocopherols, and trehalose (Tre) as potential primers that enhance the salinity tolerance of crop plants.

## Introduction

The improvement of salt tolerance of agricultural crops is indispensable to achieving future food security challenges in different regions around the world (Chourasia et al., 2022; Mangal et al., 2022). Several strategies for improving crop salt tolerance are currently in practice worldwide, including classical breeding and selection, wide crossing, transgenics, and genome editing.

Engineering crop salt tolerance has gained considerable attention during the last few decades (Farhat et al., 2019; Wani et al., 2020), but limited success has been achieved through this approach due to complex interactions and cost-intensive methodologies used (Munns and Tester, 2008; Kotula et al., 2020). Meanwhile, various alternative approaches have been employed for enhancing crop tolerance to salt stress (Zulfiqar, 2021). One of them is seeds/seedling priming. Priming, known as hardening or sensitization, could also be induced naturally or by exposure to a specific abiotic stress (Savvides et al., 2016; Liu et al., 2022a). Such priming acts as a signal, specifying an enhanced possibility of enduring a particular stress response (Filippou et al., 2013; Ibrahim, 2016; Liu et al., 2022a). After the identification of this particular stress, plants prepare themselves to respond quicker and better to their exposure to environmental stress(es) – a phenomenon called the primed state (Johnson and Puthur, 2021). The priming technique triggers the primed state in plants, which is similar to the natural priming occurring after exposure of a seed or a seedling to a specific priming agent that may be a synthetic chemical or a natural compound. As priming is believed to improve the performance of various crops under salt stress (El-Beltagi et al., 2022; Xie et al., 2022), this strategy can be a vital alternative when limited success is expected through genetic engineering.

The use of molecules with signaling properties, such as H_2_S, NO, and H_2_O_2_ or natural compounds, such as chitosan, melatonin, ascorbic acid (AsA), alpha-tocopheol, trehalose (Tre), and polyamines, and plant extracts as priming agents has been demonstrated to enhance salt tolerance in different crop plants.

This review aimed to briefly summarize key characteristics of salt stress in crop plants, introduce chemical priming agents commonly used in inducing a primed state, and present likely physiological and biochemical mechanisms involved in the primer–plant interactions. The practical use of priming agents for improving salt tolerance of different crop plants and future perspectives for exploring their potential for enhancing crop salt tolerance were also discussed.

## Salt stress: A general overview

Soil salinization is considered one of the most important environmental and agricultural issues. Globally, almost 831 million hectares of land were affected by salinization (Butcher et al., 2016). Excessive salt, mainly sodium (Na), can deteriorate soil quality and adversely impact crop productivity worldwide (Yu et al., 2020). Salt stress on crops can be further aggravated by industrial pollution and poor irrigation practices combined with the increasing human population (Hayat et al., 2020). On the other hand, the agricultural sector is under significant pressure to produce more food from saline soils. Exploiting saline–alkali lands for agriculture could provide an opportunity to fulfill the food security challenges (Yang and Guo, 2018).

Salt stress affects crop yields to varying degrees, depending on salt concentrations, crop species, stage of crop development at which the stress occurs, and stress duration (Munns et al., 2020). Saline-alkali land has high Na^+^ levels, resulting in hypertonic and hyperosmotic conditions that impede water and nutrient uptake by plants. Crops under a saline environment accumulate excessive ions in vacuole to avoid accumulation in the cytoplasm that can impair enzymatic reactions and block vital physiological and biochemical processes. However, excessive amounts of Na^+^ are lethal for plant growth. Osmotic stress is the first response of plants when salinity levels disturb the water balance. The next phase, ionic stress, increases Na^+^ ion uptake, disturbing K^+^ uptake and nutrition imbalance. Salt stress disturbs leaf water balance and ion compartmentalization (Arif et al., 2020). Osmotic and ionic stress conditions lead to oxidative stress through uncontrolled generation of reactive oxygen species (ROS), including singlet oxygen (^1^O_2_), hydroxyl radical (^⋅^OH), H_2_O_2_, and superoxide ion (O_2_^⋅–^) (Hasanuzzaman et al., 2020). Under the normal growing environment, ROS are the byproduct of several metabolic pathways that involve subcellular compartments, including chloroplasts, cytosol, mitochondria, plasma membrane, and peroxisomes (Corpas et al., 2015, 2020; Hu et al., 2020). The abundant production of ROS triggers oxidative stress, damaging crucial macromolecules (proteins, lipids, DNA, and carbohydrates), plant cellular membranes, cellular redox status, and antioxidant systems, ultimately causing cell death (Gill and Tuteja, 2010). Crops try to protect the cell from oxidative stress by activating scavenger enzymatic and non-enzymatic systems. The excessive production of ROS also disturbs the electron transport system, photosystem I (PSI), PSII system reaction centers in the thylakoid membrane, and membrane structure (Li et al., 2017). Membrane lipid oxidation has deleterious effects by inducing the formation of free radicals by “chain reaction” (Demidchik, 2015). An increase in markers related to oxidative stress, such as protein oxidation, lipid peroxidation, and 8-oxoguanine, reflects excess ROS formation that disturbs the normal functioning of cellular structures under salt stress (Møller et al., 2007; Manai et al., 2014a; Shan and Liu, 2017). Increased ROS due to salt stress also causes various metabolic alterations in protein development, membrane fluidity, blockage of electron transfers, and PSII (Biswal et al., 2011; Silva et al., 2011; Jajoo, 2013). Lipid peroxidation (LP) involves the initiation, propagation, and termination of lipids (Farmer and Mueller, 2013). During the lipid peroxidation initiation stage, one hydrogen (H) atom is excluded from the lipid molecule, resulting in a lipid radical that interacts with O_2_ to form a lipid peroxyl radical (Demidchik, 2015; Soares et al., 2019). During the propagation stage, the lipid peroxyl radical disturbs adjacent fatty acids, transferring H^+^ to form a lipid radical and lipid hydroperoxide (LOOH) (Yadav and Ramana, 2013) that damage the membranes and cause organelle degeneration and dysfunction. Malondialdehyde (MDA) is the terminal product of LP under stress (Demidchik, 2015), leading to the oxidation of histidine and lysine (Demidchik, 2015). Hence, an increased MDA in plants reflects oxidative stress (Soares et al., 2019). For instance, ElSayed et al. (2022b) reported increased MDA in zucchini in response to excess salt treatment. Numerous studies have shown that salt stress triggers LP (Shan and Liu, 2017; Shams et al., 2019).

## Improved salt tolerance by different priming agents

**FIGURE 1:**
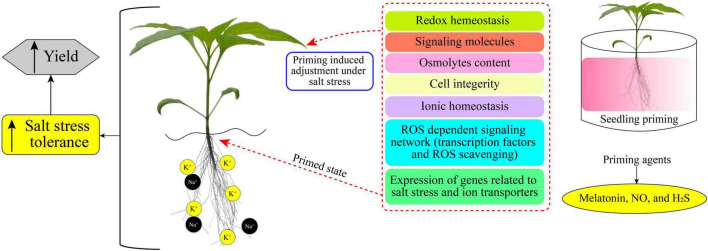
Priming induced cellular mechanisms for mediating salt-stress tolerance and yield improvement. Chemical priming (on right) can remediate salt-induced negative impacts on plants by altering metabolic processes (middle) and thus enhancing salt tolerance and yield (on left).

Plant priming is a state of plant sensitization to tolerate upcoming stress conditions (Borges et al., 2014). In a primed state, plants become ready and induced to endure the prevailing stress condition (Molassiotis et al., 2016; Marthandan et al., 2020). Various research reports have demonstrated the positive impacts and the increases in salt-stress tolerance of primed plants (Taïbi et al., 2021; El-Beltagi et al., 2022; Xie et al., 2022). Different chemicals (synthetic and natural) caused a priming state in plants, which then showed enhanced growth and increased yield under saline stress (Gohari et al., 2020a,b; Liu et al., 2022b). Figure 1 displays a simple model of plant priming agents induced plant salt tolerance. Commonly used priming agents include (1) phytohormones including brassinosteroids (BRs), ethylene, melatonin, methyl jasmonate (MeJA), salicylic acid (SA), nitric oxide (NO), and strigolactones (SLs); (2) reactive species, such as hydrogen peroxide (H_2_O_2_), hydrogen sulfide (H_2_S), and molecular hydrogen (H_2_); (3) osmoprotectants including glycine betaine, polyamines, proline (Pro), and Tre; (4) vitamins, such as alpha-tocopherol, AsA, and thiamine; and (5) mineral elements including silicon and various nanoparticles (NPs), and chitosan polymer. Biochemical, physiological, and cellular responses as well as growth and productivity improvement of different crops induced by the priming agents are presented in Table 1.

**TABLE 1:** Representative examples of beneficial effects of different compounds used for priming to improve salt tolerance in different plant species.

Plant species	Salt level	Priming agent	Major beneficial effects under salt stress	References
Pistachio (*Pistacia vera*)	120- and 240-mM NaCl	H_2_O_2_ (1, 5, and 10 mM)	• Increased total ASA and carotenoid contents. • Enhanced APX, GSH, and CAT activities.	Bagheri et al., 2019
Sweet basil (*O. basilicum*)	50-mM NaCl	H_2_O_2_ (2.5 and 5 mM)	• Improved chlorophyll and anthocyanin contents. • Enhanced GP and APX enzymes’ activities. • Improved essential oils.	Gohari et al., 2020a
Strawberry (*Fragaria* × *ananassa*)	100-mM NaCI	NaHS (100 μM)	• Increased leaf chlorophyll fluorescence, stomatal conductance, and leaf relative water content. • Minimized oxidative and nitrosative stress. • Regulated gene expression of key antioxidants, transcription factor (DREB), and salt overly sensitive (SOS) pathway genes (*SOS2-like*, *SOS3-like*, and *SOS4*).	Christou et al., 2013
Cucumber (*Cucumis sativus*)	200-mM NaCl	NaHS (20 μM)	• Alleviated reduction in photosynthetic attributes, chlorophyll fluorescence, and stomatal parameters. • Increased endogenous H_2_S level. • Increased D/L-cysteine desulfhydrase and β-cyanoalanine synthase and decreased *O*-acetyl-L-serine(thiol)lyase activities. • Maintained Na^+^ and K^+^ homeostasis by regulating the expression of *PM H-ATPase*, *SOS1*, and *SKOR* at the transcriptional level.	Jiang et al., 2019
Pakchoi (*Brassica chinensis*)	100-mM NaCl	SNP (5, 10, 25, 50, 100, and 200 μM)	• Increased germination potential, germination index, vitality index, seedling growth, SOD, POD, CAT, and APX activities, proline, K^+^/Na^+^ ratio. • Lowered MDA, O_2_^–^, and H_2_O_2_ contents.	Ren et al., 2020
Moldavian balm (*D. moldavica*)	50 and 100 mM	SNP and H_2_O_2_ (50, 100, and 200 mg L^–1^)	• Improved all agronomic traits and increased antioxidant enzyme activities. • Improved oil quality.	Gohari et al., 2020b
Basil (*O. basilicum*)	100-mM NaCl	Melatonin (1 and 10 μM)	• Improved shoot length, root length, root weight, plant weight, relative water content, photosynthetic pigments, total phenolics, and flavonoids. • Decreased DPPH antioxidant activity.	Bahcesular et al., 2020
Cucumber (*C. sativus*)	150 μmol L ^–1^ NaCl	Melatonin (50, 100, and 200 μmol L^–1^)	• Improved cell viability and protected photosynthesis. Increase antioxidant enzyme activity. • Inhibited active oxygen explosion, reduced MDA content, and relative conductivity in seedlings. • Increased the expression of antioxidant enzyme gene, nicotinamide adenine dinucleotide phosphate (NADPH) oxidase genes, mitogen-activated protein kinase (*MAPK*) genes (*MAPK3*, *MAPK4*, and *MAPK6*), and salt overly sensitive (SOS) genes (*SOS1*, *SOS2*, and *SOS3*).	Zhang et al., 2020
Cucumber (*C. sativus*)	150 mmol L^–1^ NaCl	GR24@1.0 μmol L^–1^ individually or combined with 100 μmol L^–1^ DPI (NADPH oxidase inhibitor)/40 μmol⋅L^–1^ or Ca^2+^ chelator/blocker @ 1 μmol.L^–1^	• Maintained the ion balance. • Increased the activity of antioxidant enzymes reduced the membrane lipid peroxidation. • Increased the activities of CAT, POD, and APX. • Decrease relative conductivity. • Increase in the proline content. • Elevated gene expression levels of antioxidant enzymes, nicotinamide adenine dinucleotide phosphate (NADPH) oxidase, calcium-dependent protein kinases (CDPKs), salt overly sensitive SOS1, CBL-interacting protein kinase 2 (CIPK2), and calcineurin B-like protein 3 (CBL3).	Zhang et al., 2022a,b
Sunflower (*Helianthus annuus*)	120-mM NaCl	Strigolactones (0.001, 0.01, and 0.1 mg L^–1^)	• Enhanced plant biomass and water use efficiency. • Reduced Na^+^ content in roots and shoots.	Sarwar and Shahbaz, 2020
Cucumber (*C. sativus*)	150 mmol L^–1^ NaCl	Strigolactones (1.0 μmol L^–1^)	• Increased chlorophyll contents, stomatal conductance, greater efficiency of photosynthetic. • Alleviated salt-induced photodamage, enhanced the efficiency of ascorbate–glutathione (AsA–GSH) cycle, and scavenged excessive ROS, thereby alleviating oxidative. • Differentially expressed genes related to MAPK cascade pathway, photosynthesis and oxidation, and antioxidant system.	Zhang et al., 2022b
Quinoa (*Chenopodium quinoa*)	400-mM NaCl	Saponin (0.5, 2, 5, 10, 15, 25, and 35%)	• Improved seed germination. • Elevated photosynthesis rate, stomatal conductance, osmotic and water potentials, K^+^, and abscisic acid. • Decreased Na^+^ ions.	Yang et al., 2018
Wheat (*Triticum aestivum*)	6 and 12 dS m^–1^	Salicylic acid (75 mg L^–1^) individually or along with 70 mg L^–1^ melatonin.	• Increased N, P, K, Fe, Zn, and Cu acquisition, accompanied by significantly reduced Na^+^ accumulation. • Enhanced ATP content and H^+^-pump activity of roots. • Increased antioxidants (SOD, POX, CAT, and polyphenol oxidase).	Talaat and Shawky, 2022
Barley (*Hordeum vulgare*)	150- and 300-mM NaCl	Salicylic acid (0.5 mM)	• Increased leaf proline content. • Enhanced indole-3-acetic acid. • Decreased ABA, JA, ethylene, and most of CKs.	Torun et al., 2022
Lentil (*Lens culinaris*)	75 and 150 mM	Salicylic acid (0.1, 0.5, and 1 mM) H_2_O_2_ (0.05, 0.1, and 0.120 mM).	• Increased germination percentage, root elongation, total lipids, and guaiacol peroxidase activity. • Decreased MDA.	Bouallègue et al., 2019
Maize (*Zea mays*)	100-mM NaCl	Cerium oxide NPs (1, 5, 10, 20, and 50 mg L^–1^)	• Maintained Na^+^/K^+^ homeostasis. • Enhanced photosynthetic efficiency. • Decreased ROS level. • Antioxidative defense system-related genes recovered to those of normal control level. • Enhanced yield.	Liu et al., 2022a,b
Valencia’ sweet orange (*Citrus* × *sinensis* L. Osbeck)	60- or 120-mM NaCl	Silicon NPs (200, 400, and 600 mM)	• Improved root growth and chlorophyll content. • Enhanced Na^+^ cotransporter and aquaporin transcripts in root tissues.	Mahmoud et al., 2022
Tomato (*S. lycopersicum*)	50-mM NaCl	Copper NPs (250 mg L^–1^)	• Improved Na^+^/K^+^ ratio. • Increased APX, PAL, GPX, SOD, and CAT activities.	Pérez-Labrada et al., 2019
Mung bean (*V. radiata*)	1-, 10-, 20-, and 50-mM NaCl	Sodium silicate as silicon source (1 and 5 mM)	• Increased the expression of photosynthetic proteins such as PSI, PSII, and light harvesting complex proteins. • Improved photosynthetic pigments and leaf gas exchange characteristics. • Improved formation of root nodules. • Enhanced proline accumulation. • Decreased Na^+^ uptake and increased K^+^ uptake.	Al Murad and Muneer, 2022
Maize (*Z. mays*)	80- and 160-mM NaCl	Silicon (1 mM)	• Enhanced SOD, POD, APX, and CAT activities. • Decreased Na^+^/K^+^ ratio, and Na^+^ ion uptake at the surface of maize roots and translocation in plant tissue.	Ali et al., 2021
Lettuce (*Lactuca sativa*)	100-mM NaCl	Chitosan (100 mg L^–1^)	• Increased leaf chlorophyll a, proline, and soluble sugar contents; and POX and CAT activities, and alleviated membrane lipid peroxidation.	Zhang et al., 2021
Safflower (*C. tinctorius*) and sunflower (*H. annuus*)	3.4, 6.1, 8.6, and 10.8 dS m^–1^	Chitosan (0.25, 0.50, and 0.75%)	• Increased germination percentage. • Decreased MDA and proline contents; and CAT and POX activities.	Jabeen and Ahmad, 2013
Tomato (*S. lycopersicum*)	50-, 100-, 150-, and 200-mM NaCl	Trehalose (5, 10, and 25 mM)	• Improved chlorophyll fluorescence traits. • Increased the contents of osmotic substances, carbohydrates, K^+^, and K^+^/Na^+^ ratio. • Promoted trehalose metabolism. • Elevated antioxidant enzyme activities (SOD, POD, and CAT) and expression of related genes (*SlCu/Zn-SOD*, *SlFe-SOD*, *SlMn-SOD*, *SlPOD*, and *SlCAT*).	Xie et al., 2022
Strawberry (*Fragaria* × *ananassa*)	50-mM NaCl	Trehalose (10 or 30 mM)	• Helped maintained photosynthetic electron transport rate. • Reduced shoot Na^+^ accumulation. • Improved carotenoids, flavonoids, and anthocyanins.	Samadi et al., 2019
Rapeseed (*B. napus*)	150 NaCl	Spermine (0.25 mM) or Spermidine (0.25 mM)	• Elevated chlorophyll and proline contents. • Maintained higher photosystem II (PSII) activity. • Improved CO_2_ assimilation and significantly elevated rubisco activity (ribulose 1,5-bisphosphate carboxylase/oxygenase). • Enhanced the polyamine pathway as observed by upregulated transcription of polyamine oxidase (*PAO*) and diamine oxidase (*DAO*).	ElSayed et al., 2022a
Calendula (*C. officinalis*)	1, 5, and 9 dS m^–1^	Spermine and spermidine (both at 0, 0.5, and 1 mmol L^–1^)	• Improved proline and protein contents. • Improved total chlorophyll. • Improved POD and CAT. • Increased maximum photochemical quantum yield (*F*_*V*_/*F*_*m*_).	Baniasadi et al., 2018
Pea (*Pisum sativum*)	150-mM NaCl	Thiamine (0, 250, and 500 mg L^–1^)	• Improved total soluble sugars. • Enhanced proline and phenolics.	Naheed et al., 2022
Faba bean (*Vicia faba*)	1.37, 3.31, and 4.51 dS m^–1^ NaCl	Thiamine (0, 50, 75, and 100 mg L^–1^)	• Enhanced polysaccharides, total carbohydrates, free amino acids, and proline. • Enhanced growth and yield.	El-Metwally and Sadak, 2019
Wheat (*T. aestivum*)	100-mM NaCl	Ethylene (200 μL L^–1^ ethephon)	• Higher growth and photosynthesis through a reduced Glu sensitivity. • Ethylene-induced reduced glutathione (GSH) production resulted in increased psbA and psbB expression to protect PSII activity and photosynthesis.	Sehar et al., 2021
Mustard (*B. juncea*)	100-mM NaCl	Ethylene (25 ml of 200 μl L^–1^ ethephon)	• Enhanced photosynthetic efficiency by increasing the assimilation of N and S, improving the concentration of proline and induction of the antioxidant system. • Reduced oxidative stress.	Jahan et al., 2021
Lettuce (*L. sativa*)	15-mM NaCl	Proline (0, 5, 10, and 15 μM)	• Improved photosynthesis. • Increased salt-stress tolerance.	Orsini et al., 2018
Maize (*Z. mays*)	80-mM NaCl	Proline (30 mM)	• Reduced Na^+^ and Cl^–^ accumulation together with increased K^+^ content. • Enhanced CAT, APX, and SOD activities. • Reduced oxidative stress.	de Freitas et al., 2018
Common bean (*P. vulgaris*)	50 and 100 mM NaCl	Glycine betaine (25- and 50-mM GB at a rate of 50 ml per plant)	• Increased the antioxidant defense including both enzymatic (i.e., peroxidase, superoxide dismutase, and catalase) and non-enzymatic (i.e., proline and glutathione) agents. • Reduced the accumulation of Na^+^ and at the same time induced K^+^ uptake maintaining a higher K^+^/Na^+^ ratio.	Sofy et al., 2020
Cotton (*Gossypium hirsutum*)	150 mM NaCl	Glycine betaine (5 ml per plant)	• Increased leaf gas exchange. • Decreased the carboxylation efficiency (*P*n/*C*i) and water use efficiency (WUE).	Hamani et al., 2021
Arabidopsis (*Arabidopsis thaliana*)	35-mM NaCl	Hydrogen (1.0 μM)	• Lowered Na^+^/K^+^ ratios and higher levels of ion transport–related gene transcripts. • Improved chlorophyll contents and Pro concentration. • Altered melatonin signaling.	Su et al., 2020
Rapeseed (*B. napus*)	150-mM NaCl	Ammonium borane-based hydrogen-rich water (2 mg L^–1^ NH3⋅BH3-prepared HRW)	• Decreased Na content and increased K content, resulting in a decreased Na/K ratio. • Increased proline accumulation *via* altering enzymes related to it.	Zhao et al., 2021
Okra (*A. esculentus*)	100-mM NaCl	Alpha-tocopherol (200 and 300 mg L^–1^)	• Increased activities of antioxidants (CAT, GPX, and SOD) and levels of ascorbic acid, accumulation of GB, and total free proline • Reduced Na^+^, MDA, and H_2_O_2_ levels • Improved the uptake of K^+^ and Ca^2+^.	Naqve et al., 2021
Common bean (*P. vulgaris*)	EC 6.35–6.42 dS m^–1^	Alpha-tocopherol (1 mM)	• Improved mineral nutrients (N, P, K, and Ca), and osmoprotectants (soluble sugars, and proline), non-enzymatic (ascorbic acid, glutathione, and TOC) and enzymatic [superoxide dismutase, catalase, and guaiacol peroxidase (GPOX) antioxidants].	Hemida et al., 2017
Barley (*H. vulgare*)	200 mM NaCl	Ascorbic acid (200 mg L^–1^)	• Enhanced vegetative growth. • Improved photosynthetic pigments, and synchronized ion uptake. • Elevated synthesis of enzymatic and non-enzymatic antioxidants, and the harvest index.	Noreen et al., 2021
Calendula (*C. officinalis*)	25-, 50-, 75-, and 100-mM NaCl	Ascorbic acid (0, 3, and 6 mM)	• Decreased proline content, and cell membrane injury. • Increased water content. • Decreased antioxidant activity.	Azizi et al., 2021
Cucumber (*C. sativus*)	150 mmol L^–1^ NaCl	Strigolactones (1.0 μmol L^–1^)	• Upregulated H_2_O_2_ and MAPK cascade pathway and differentially expressed genes (DEGs). • Alleviated salt-induced photodamage, enhanced the efficiency of ascorbate–glutathione (AsA–GSH) cycle, and scavenged excessive ROS.	Zhang et al., 2022b
Rice (*Oryza sativa*)	200 mM NaCl	Strigolactones (0.1, 0.2, 1, and 2 μM)	• Improved salt-stress tolerance *via* elevated antioxidant activities. • Reduced MDA content.	Ling et al., 2020
Basil (*O. basilicum*)	30-, 60-, and 90-mM NaCl	Methyl jasmonate (0 and 0.5 mM)	• Enhanced the levels/activities of antioxidants. • Improved volatile oil.	Talebi et al., 2018
Sea fennel (*Crithmum maritimum*)	150-mM NaCl	Methyl jasmonate (0.5 mM)	• Maintained the antioxidant nutritional properties. • Increased total flavonoids.	Labiad et al., 2021

SNP, sodium nitroprusside; NaHS, sodium hydrosulfide; NaCl, sodium chloride; DPHH, 2,2-diphenyl-1-picrylhydrazyl; POD, peroxidase; CAT, catalase; SOD, superoxide dismutase; POX, peroxidase; H_2_S, hydrogen sulfide; AsA, ascorbic acid; MDA, malondialdehyde; H_2_O_2_, hydrogen peroxide; PAL, phenylalanine ammonia lyase; APX, ascorbate peroxidase; GPX, glutathione peroxidase; Cu, copper; GSH, glutathione; GP, guaiacol peroxidase; ROS, reactive oxygen species.

### Phytohormones

#### Brassinosteroids

BRs, a group of plant steroids and polyhydroxylated hormones play a vital role in the growth and developmental activities of plants (Gruszka, 2013; Kong et al., 2021). Accumulating evidence shows that the pretreatment with BRs triggered salinity stress resistance in different plant species. For example, Su et al. (2020) reported that the BR application on *Malus hupehensis* under salt stress eliminated ROS production and improved superoxide dismutase (SOD, E.C. 1.15.1.1) and catalase (CAT, E.C. 1.11.1.6), Pro, soluble sugars, and K^+^ contents. Further, the Na^+^ uptake decreased *via* regulating *MhNHXs* [Na^+^ (K^+^)/H^+^ antiporter genes]. Yue et al. (2020) compared the seed priming and seedling priming with 24-epibrassinolide on black locusts (*Robinia pseudoacacia* L.) under salt stress. The authors observed that primed seedlings performed comparatively better on mediating salt tolerance by improving chlorophyll content, leaf gas exchange, and membrane stability while decreasing leaf Na^+^ level and oxidative stress markers, such as H_2_O_2_ and MDA.

The current understanding of BRs mediated stress responses is largely based on the Arabidopsis BR signaling model. The BRI1 (BRASSINOSTEROID INSENSITIVE 1) and BAK1 (BRI1-ASSOCIATED RECEPTOR KINASE 1) as the BRI-BAK1 receptor complex located on the plasma membrane perceives BRs, and the signaling is sequentially transduced by BSK1 (BR-SIGNALING KINASE 1), BSU (BR-SUPPRESOR 1) phosphatase, and BIN2 (BRASSINOSTEROID-INSENSITIVE 2 kinase), which result in the activation of BZR1 and BES1 (BRASSINAZOLE-RESISTANT 1 and BRI1-ETHYLMETHYLSULFONE-SUPPRESSOR 1), two key transcriptional factors of BR signaling (Kim and Wang, 2010). Each of these components is a family of protein members and participates in the regulation of stress tolerance (Ahammed et al., 2020). For example, BAK1 belongs to SERK (SOMATIC EMBRYOGENESIS RECEIPTOR-LIKE kinase) family proteins. The SERK2 can interact with OsBRI1 in rice, and overexpression of SERK2 substantially increased salt tolerance and grain size (Dong et al., 2020). Additionally, exogenous supplementation of BR can induce the expression of RESPIRATORY BURST OXIDASE HOMOLOG 1 (RBOH1) encoding NADPH oxidase which causes ROS generation in the apoplast. Further BR-induced ROS signals moderate redox homeostasis, triggering activation of transcription factors (TFs) that manage transcription of BR-regulated and stress-responsive genes. It ultimately leads to improved resistance to abiotic stresses *via* an increase in protective protein controls.

#### Ethylene

Ethylene is a gaseous plant hormone that has also a crucial role in plants’ adaptation under stress conditions. It particularly regulates the photosynthesis process under salinity stress (Fatma et al., 2021; Rasheed et al., 2021). Borbély et al. (2020) evaluated the response of exogenous ethylene on salt-stressed tomato plants, observed a reduction in salt-induced osmotic effects, and regulated carbon assimilation and photosynthetic traits and non-photochemical quenching (NPQ) through regulating starch metabolism and PSI cyclic electron flow in tomatoes. Jahan et al. (2021) evaluated the combined application of ethylene together with sulfur and nitrogen on salt-stressed mustard (*Brassica juncea*) and observed an improved salt-stress tolerance through the regulation of pro-metabolism and antioxidants. Fatma et al. (2021) inspected the combined effect of sulfur and ethylene on salt-stressed mustard, observed the application improved ABA and the development of chloroplast thylakoids and photosynthetic performance, reduced oxidative stress *via* non-enzymatic and enzymatic components of ascorbate–glutathione (AsA–GSH) cycle, and decreased H_2_O_2_ and Na ions uptake.

#### Melatonin

Melatonin is a biological indoleamine molecule regulating numerous vital processes in plants (Altaf et al., 2021, 2022; Arnao and Hernández-Ruiz, 2021; Zhao et al., 2022). Different studies have showed that the pretreatment with melatonin positively affects plants under salinity. ElSayed et al. (2020) described that pretreatment of peanut (*Arachis hypogaea*) with melatonin improved salt stress *via* reduction of ROS. Further, pretreatment to salt-stressed *A. hypogaea* upregulated melatonin biosynthesis-associated genes, such as *ASMT1*, *ASMT2*, *ASMT3*, and *TDC*. Furthermore, tryptamine 5-hydroxylase (T5H) and redox homeostasis were also influenced by the ability of melatonin to induce alterations in antioxidant systems, such as increases in the expressions of *APX, CAT, SOD, GR*, and *DHAR* encoding genes. The application of melatonin on roots of watermelon seedlings grown under salinity stress, improved growth, increased photosynthesis, and decreased oxidative stress by improving the redox state coupled with enhanced antioxidant activities (Li et al., 2017). Gao et al. (2019) reported that the improved growth following melatonin application was linked with oxidative stress reduction and upregulation of genes encoding *chlorophyll synthase* (ChlG), *lipid peroxidase, lipoxygenase*(LOX), and *peroxygenase*. Altaf et al. (2021) reported that melatonin application on salt-stressed tomato (*Solanum lycopersicum* L.) plants ameliorated the negative impact by changing root system architecture and by increasing photosynthetic pigments and assimilations. The authors further explained that this was caused by the obstruction of Na uptake from soils and transport to the aerial parts of the plant and the reduction of the oxidative stress justified by the decrease in O_2_^⋅–^, H_2_O_2_, electrolytes leakage, and MDA. Moreover, melatonin is also associated with boosting antioxidant enzymes and compounds, such as ascorbate peroxidase (APX E.C. 1.11.1.11), SOD, CAT, AsA, glutathione reductase (GR E.C. 1.8.1.10), and GSH, improving cellular membrane stability. While working with salt-stressed strawberry (*Fragaria* × *ananassa* Duch., cv. “Camarosa”) Zahedi et al. (2020) observed that melatonin application not only revived growth but also improved fruit yield and quality. Liu et al. (2022b) examined the impact of melatonin on salt-stressed sugar beet (*Beta vulgaris* L.) and observed that the application of melatonin improved salt tolerance by activation CAT, SOD, peroxidase (POD, E.C. 1.11.1.7) and reduction of H_2_O_2_ and MDA production. In purple-leaved basil (*Ocimum basilicum* L.), seed priming with melatonin was able to reduce the negative effect of salinity as demonstrated by the metabolites and antioxidant compounds changes (Bahcesular et al., 2020).

Zhang et al. (2020) examined the effect of melatonin on the salt-stress tolerance mechanisms of cucumber. The authors observed that melatonin application improved photosynthesis, cell viability, and antioxidant enzyme activity and reduced MDA content, relative conductivity, and active oxygen explosion in cucumber seedlings. Gene expression analysis in the same study showed that melatonin application in salt-stressed cucumber enhanced the expression of nicotinamide adenine dinucleotide phosphate (NADPH) oxidase genes, antioxidant enzyme gene, *salt overly sensitive* (*SOS*) genes, and *mitogen-activated protein kinase* (*MAPK*) genes. SOS are genes that regulate the Na^+^ transporters through the membrane and can regulate the Na^+^ concentration in the cytoplasm. The activation of SOS genes is mediated by intracellular calcium concentration.

All these reports show that melatonin has a positive outcome for plant priming by regulating the accumulation of antioxidants and endogenous melatonin during salt stress. The molecular mechanism of melatonin-induced alleviation of salt stress still needs to be explored, and the genes related to antioxidants and ions transporters regulated by melatonin need to be determined in more crops.

#### Methyl jasmonate

MeJA is a gas volatile organic compound and plant growth regulator and plays an integral role in the regulation of tolerance mechanisms against environmental stresses. The application of MeJA has been observed to enhance salt-stress resistance in different crop plants. For instance, Ahmadi et al. (2018) evaluated the effect of exogenous MeJA on *B. Napus* and reported mitigation of salt stress *via* increasing water content, photosynthesis rate, and soluble sugar content. In salt-stressed sea fennel (*Crithmum maritimum* L.), Labiad et al. (2021) observed that MeJA improved salt-stress responses by maintaining antioxidant and flavonoid production. While working with salt-stressed sweet basil, Talebi et al. (2018) reported an increase in oil quality and antioxidants that lead to relief from salt stress in sweet basil. In response to MeJA treatment, Gao et al. (2021) observed an improvement in salt-stress tolerance in *Nitraria tangutorum* Bobrov by increasing the accumulation of osmolytes, antioxidant activity, and antioxidant content and lowering Na^+^/K^+^ ratios in shoots and higher Na^+^ efflux rates in roots of plants. Furthermore, MeJA augmented endogenous ABA and JA, and the transcript levels of their biosynthesis and responsiveness genes (Gao et al., 2021). In salt-stressed *Citrus sinensis* (L.) Osbeck, the application of MeJA promoted growth, photosynthetic pigment, APX1, CAT, CSD, GSTs, POD, PAL, Na^+^ co-transporters, and aquaporin proteins (Mahmoud et al., 2021). Lang et al. (2020) reported that MeJA mediates salt-stress tolerance in *Glycyrrhiza uralensis* Fisch. Ex DC. *via* the regulation of antioxidative defense, and carbon and nitrogen metabolism. Moreover, MeJA is also reported to provide salt-stress tolerance in strawberry *via* altering the expression of stress-associated genes (Moradi et al., 2022).

#### Nitric oxide

NO is known for its vital role in plant cellular signaling (Sun et al., 2021). It participates in various processes of plant growth under both normal and unfavorable conditions (Corpas and Barroso, 2015; Asgher et al., 2017; Begara-Morales et al., 2018; Kolbert et al., 2019). Various studies have demonstrated the central mediating effect of NO signaling in inducing salinity tolerance (Molassiotis et al., 2010; Manai et al., 2014b). For instance, Gao et al. (2022) studied the impact of NO in mitigating the ill effects of salinity stress on *N. tangutorum*. They observed that NO relieved salt stress *via* decreasing leaf senescence and root damage while enhancing endogenous NO, activating the ascorbate–glutathione (AsA–GSH) cycle, and increasing antioxidant enzymes’ activities and the expression of their associated genes, ultimately relieving salt-stress-induced oxidative damage. Moreover, the exogenous NO promoted ion transporter and Na^+^ efflux gene expression and reduced the Na^+^/K^+^ ratio (Gao et al., 2022). Treatment of spinach (*Spinacia oleracea* L.) and tomato (*S. lycopersicum* L.) with NO triggered salt-stress tolerance *via* increasing the biosynthesis of total phenolic content, ascorbate, GSH, flavonoids, and Pro (Hayat et al., 2012; Du et al., 2015). A proteomic study performed by Shen et al. (2018) revealed that under salt stress, NO regulates the accumulation of photosynthesis-associated proteins. They observed that in salt-stressed mangrove plant *Avicennia marina* (Forssk.) Vierh., the abundance of rubisco large subunit (RBCL), ribulose-phosphate 3-epimerase, quinine oxidoreductase-like protein isoform 1 (QOR1) and rubisco activase A decreased, while the abundance of other proteins, such as RBCL and QOR1 increased by NO application (Shen et al., 2018). Additionally, exogenous NO treatment enhanced salt tolerance *via* enhancing the accumulation of protein related to primary metabolism, RNA transcription, energy metabolism, and stress response proteins (Shen et al., 2018).

In pepper (*Capsicum annuum* L. cv. Alova), NO application ameliorated salt stress *via* the regulation of leaf gas exchange traits, mineral uptake, and osmotic potential. Furthermore, the application of NO decreased MDA and H_2_O_2_ resulting in better growth and reduced salt-induced oxidative stress (Shams et al., 2019). Ahanger et al. (2020) demonstrated that exogenously applied NO and salicylic acid (SA) prompted salt-stress tolerance in *Vigna angularis* (Willd.) *via* increasing Pro, glycine betaine, and sugars, while upregulated enzymatic and non-antioxidant production, and content of N, P, and Ca. With respect to respiratory terminal oxidase which is responsible for salt-stress tolerance *via* catalyzing cyanide-resistant respiration, Jian et al. (2016) revealed that exogenous NO boosts the expression of alternative oxidase (AOX) genes and the cyanide-resistant respiration rate, which are induced by salinity stress, to relieve the salt-stress-induced photosynthetic and oxidative damage in plants. Huang et al. (2020) showed that exogenous NO treatment controls the expression of NH_4_^+^ transporters in order to mediate NH_4_^+^ transport, which may imitate N uptake and sugar transport rate and its subsequent utilization for mitigation of salt-induced oxidative stress. It is considered that programmed cell death (PCD) under salt-stress maintains cellular homeostasis and Sami et al. (2021) observed a decrease in PCD in salt-stressed mustard plants following NO supply. In addition, NO signaling with small active substances, such as polyamines, calcium ions, sulfur, and hormones is associated with increased salt-stress resistance in plants. For instance, Tailor et al. (2019) observed that NO upregulates polyamines biosynthetic enzymes, including *S*-adenosylmethionine decarboxylase and arginine decarboxylase, while it lowered the activity of polyamine oxidase to prevent its degradation, hence improving salt-stress tolerance in sunflower. Regarding NO and melatonin signaling, Zhao et al. (2021) showed that the NO and melatonin combined application promotes salt-stress tolerance *via* ion homeostasis, re-established redox and mitigation of ROS as well as modulating the salt overly sensitive 2 (*SOS2*), antioxidant defense genes, and sodium hydrogen exchanger (*NHX1*) transcripts. Sulfur (S) and NO are observed to work synergistically against salt stress. For instance, in salt-stressed mustard applications of NO and S decreased the Na^+^ and Cl^–^ uptake *via* regulating Na^+^ transporter and H^+^ pump function in both roots and leaves. Moreover, the application of NO and S to salt-stressed mustard plants encouraged the activity of ATP-sulfurylase (ATPS, EC 2.7.7.4), CAT, APX, and GR, hence reducing salt-stress-induced oxidative stress (Fatma et al., 2016). A link between NO and phytohormones regarding salt-stress tolerance in plants has been shown; for example, while observing the integrative role of auxins and NO in salt-stressed *Arabidopsis*, NO function was found to be upstream of auxins (Liu et al., 2015). The authors also observed that NO dropped auxin levels *via* suppressing the expression of the auxin efflux transporter gene PINFORMED (PIN) and decreasing auxin signal transduction through stabilizing the Aux/IAA suppressor protein IAA17, thereby resulting in restricted root meristem growth. Regarding GA and NO, Chen et al. (2022) reported that NO negatively regulates GA signaling *via* sulfhydryl nitrosylation (*S*-nitrosylation) of DELLA proteins to synchronize the plant growth under salt stress. Kong et al. (2016) noted that the foliar application of NO to salt-stressed cotton increased the expression of cytokinin biosynthesis-related genes including *IPA*, *ZR*, and *IPT1*, suggesting the vital role of NO in promoting CK biosynthesis, ultimately lowering salt-stress-induced leaf senescence. Ahmad et al. (2018) observed that NO and jasmonic acid upregulated osmolyte synthesis, antioxidant metabolism, and metabolite accumulation in salt-stressed tomato.

The above-mentioned studies implied that plant priming with NO showed better salt tolerance in crop plants by regulating the anti-oxidative defense system, nutrient uptake, photosynthesis and stress-related proteins, and osmolyte accumulation. However, there is still a need to evaluate NO induced salt tolerance mechanisms, especially anti-oxidative defense responses at the molecular level.

#### Salicylic acid

SA is a well-known phenol-based plant hormone regulating various vital endogenous processes and signals in plants. SA has been shown to induce different levels of resistance against biotic and abiotic stresses (Ding and Ding, 2020; Kaya et al., 2020; Sharma et al., 2020). The recent pieces of evidence have revealed that plant priming with SA improved plant salt-stress tolerance. According to Bukhat et al. (2020), salt stress was significantly mitigated in radish (*Raphanus sativus* L.) after the SA application. The SA-treated radish showed improved photochemistry and antioxidant activity while lowering membrane damage and ROS generation. The foliar application of 0.5-mM SA improved the growth, essential oil, and chlorophyll contents of *Salvia officinalis* L. Meanwhile, SA decreased Na content and increased Ca, K, and P contents in the leaves and roots of *S. officinalis* (Es-sbihi et al., 2021). In cucumber seedlings, the application of SA improved the relative growth rate, root growth traits, and leaf photosynthetic traits under salt-stress conditions. Furthermore, SA application downregulated *GL2* and *RHD2* and upregulated the *RHD2*, *NAC1*, *NAC2*, *GL2*, and *EXP* in salt-stressed seedlings (Miao et al., 2020). Ma et al. (2017) tested the influence of SA plant priming and reported enhanced salt-stress tolerance of *Dianthus superbus* L. through regulating photosynthesis and antioxidant systems. SA-priming is a strategy for increasing major GSH-based H_2_O_2_-metabolizing enzymes, such as glutathione-*S*-transferase (GST E.C., 2.5.1.18) (Khan et al., 2015). SA improved salt tolerance of *S. lycopersicum* through the expression of GST-gene family members including *SlGSTT2, SlGSTT3*, and *SlGSTF4* (Csiszár et al., 2014). Jayakannan et al. (2015) proposed that the SA receptor, a non-expresser of PR protein 1 (NPR1) could be a leading regulatory protein implicated in SA-based defense responses in plants. NPR1 may enhance root H^+^-ATPase activity, control Na^+^ entry into roots and subsequently transport to shoots, prevent K leakage resulting from Na stress, and increase K content in plant shoots. Our understanding of SA-mediated tolerance to salt stress is not conclusive. SA as a signal molecule may induce a large number of gene expressions in salt-stressed plants, including NPR1, K transporters, and genes related to the ROS scavenging pathways (Saleem et al., 2021). Subsequently, the activities of enzymatic antioxidants such as APX and CAT increased; and ROS and MDA are reduced, resulting in the protection of cell membrane, sustaining plant photosynthesis, and improving plant growth.

#### Strigolactones

SLs, being a new class of phytohormones, play vital roles in plant growth and productivity under normal and harsh conditions. Their role in enhancing plant salt-stress tolerance is quite promising. For instance, Zhang et al. (2022a) evaluated the exogenous applications of SLs on salt-stressed cucumber seedlings. The authors observed that the application of SL alleviated salt-stress damage by regulating photosynthesis, enhancing the efficiency of ascorbate–glutathione (AsA–GSH) cycle, and scavenging excessive ROS. Strigolactones boosted antioxidant activity and maintained osmotic and ionic balance in salt-stressed cucumber plants. Strigolactones raised the gene expression levels of nicotinamide adenine dinucleotide phosphate (NADPH) oxidase, antioxidant enzymes, calcium-dependent protein kinases (CDPKs), CBL-interacting protein kinase 2 (CIPK2), SOS1, and calcineurin B-like protein 3 (CBL3) (Zhang et al., 2022b).

### Reactive agents

#### Hydrogen peroxide

Hydrogen peroxide was used to be considered a toxic molecule, but now it has been known as an important signaling molecule with crucial roles in signal transduction pathways (Gohari et al., 2020a,b; Nazir et al., 2020). Several studies have shown that treatment of H_2_O_2_ can prompt plant tolerance to salt stress. For example, foliar-fed pistachio seedlings with H_2_O_2_ displayed enhanced salt tolerance, which was associated with the production of AsA. The treatment with H_2_O_2_ induced glutathione (GSH) and carotenoids and upregulated enzymatic antioxidants, such as CAT and APX (Bagheri et al., 2019). Salt tolerance of *Vigna radiata* (L.) R. Wilczek (mung bean) was improved by the application of H_2_O_2_ to seedlings (Shan and Liu, 2017) (Table 1). The improved salt tolerance of the primed *Vigna radiata* plants was attributed to attenuated electrolyte leakage (EL) and increased total ascorbate and GSH content, as well as increased APX, dehydroascorbate reductase (DHAR, E.C. E1.6.5.4), GR, and γ-glutamylcysteine synthetase (E.C. 6.3.2.2) activities in leaves. Likewise, Gohari et al. (2020a) tested the individual and combined effects of H_2_O_2_ and sodium nitroprusside (SNP, donor of NO) as priming agents on *O. basilicum* L. plants grown under salt stress. The authors noted that the foliar application of 2.5 mM H_2_O_2_ plus 200 μM SNP effectively improved the growth of *O. basilicum* under salt stress *via* increasing chlorophyll, carotenoid, and anthocyanin and the activities of both APX and guaiacol peroxidase. Tanou et al. (2009) noted that pre-treatments of citrus root (*Citrus aurantium* L.) with SNP and H_2_O_2_ under salt stress induced a strong antioxidant enzymatic defense response in leaves by increasing SOD, CAT, APX, GR, and related isoform(s) expression. Furthermore, it was found that the salt-induced protein carbonylation (an oxidative damage marker) was reversed and the ascorbate redox status was recovered in the pre-treated citrus plants.

Priming with H_2_O_2_ alone or in combination with other potential priming chemicals enabled plants to become tolerant to salt stress particularly by modulating several key components of the oxidative defense system. However, from these reports, it is not evident how far this priming agent triggers physio–biochemical processes other than those of the oxidative defense system, involved in the salt tolerance mechanism.

#### Hydrogen sulfide

Hydrogen sulfide (H_2_S) is a highly soluble, colorless, and flammable gas (Calvert et al., 2010). In plants, H_2_S is a gas transmitter similar to NO, playing a crucial role in plant vital processes and in the alleviation of various biotic and abiotic stresses (Hancock and Whiteman, 2016; Corpas et al., 2019; Zulfiqar and Hancock, 2020; Cheng et al., 2022). Furthermore, H_2_S is considered a reactive molecule for cell signaling events in plants (Corpas et al., 2019; Hancock, 2019). It has been reported to mediate salinity tolerance *via* the regulation of various physiological and biochemical responses (Shan et al., 2014; Ma et al., 2019). For example, Christou et al. (2013) tested H_2_S as a potent priming agent for strawberry (*Fragaria* × *ananassa* cv. Camarosa) plants grown under salt stress. They noted that pretreatment of roots with sodium hydrosulfide (NaHS) (a H_2_S donor) increased stomatal conductance and leaf relative water content and decreased salt-induced nitrosative and oxidative damage in plants. Furthermore, gene expression analysis revealed that H_2_S induced various transcriptional changes related to antioxidants and the salt overly sensitive (SOS) pathway. Jiang et al. (2019) reported that H_2_S priming induced salt tolerance in cucumber (*Cucumis sativus* L.) cultivar ‘Chunxiaqiuwan’ plants was related to the improved stomatal traits, photosynthetic pigments, and enhanced endogenous H_2_S levels. Hydrogen sulfide priming induced β-cyanoalanine synthase (E.C. 4.4.1.9) and D/L-cysteine desulfhydrase (E.C. 4.4.1.15) activities and reduced *O*-acetyl-L-serine (thiol)lyase. Furthermore, under higher NaCl levels, H_2_S maintained homeostasis of the ions Na^+^ and K^+^ by regulating PM H^+^-ATPase, SKOR, and SOS1 at the transcriptional level (Jiang et al., 2019).

Overall, the current understanding of H_2_S-mediated plant salt tolerance is largely linked with the regulation of physiological traits, ion balance, and enhanced antioxidant activities that may prevent oxidative damage associated with salinity stress. Further studies are compulsory to enhance the understanding of the salt-stress mitigation phenomenon by H_2_S plant priming especially focusing on the molecular aspects under field experiments.

#### Molecular hydrogen

The application of hydrogen (H_2_) gas to plants in the form of hydrogen-rich water (HRW) or H_2_-saturated water is a new method for boosting the ability of plants against the ill effects of abiotic stresses (Zulfiqar et al., 2021). Currently, H_2_ has been tested on different crop plants for its ability to impart salt-stress tolerance. For instance, Yu et al. (2021) tested the impact of HRW on salt-stressed cucumber plants. The authors observed a marked improvement in the salt-stress tolerance in cucumber plants following the applications of HRW. In another study with *B. napus*, Zhao et al. (2021) examined the role of HRW under different stresses including salt stress. The authors observed that the applications of HRW on salt-stressed brassica could ameliorate the negative impact of salt stress *via* regulating ion and redox balance through the involvement of NO. The hydrogen-rich water applications decreased the Na/K ratio in rapeseed seedlings grown under salt stress (Zhao et al., 2021).

Studies are limited related to the HRW applications on crop plants, and hence further research particularly focusing on the molecular aspect is necessary to better understand the HRW-mediated salt-stress tolerance in plants.

### Osmoprotectants

#### Glycine betaine

Glycinebetaine (GB) is considered a vital osmoprotectant in plants. Glycinebetaine is involved in the mitigation of adverse effects associated with abiotic stresses including salt stress in plants. Exogenous supplementation of GB is reported to enhance the resistance to salt stress in plants. Yildirim et al. (2015) evaluated the role of exogenous GB on lettuce and reported that the GB applications reduced membrane permeability, MDA, H_2_O_2_, and Na accumulation while raising the concentrations of indole acetic acid (IAA), SA, and GA; hence, imparting salt-stress tolerance to lettuce plants. In salt-stressed common beans, the application of GB reduced Na uptake and boosted antioxidant activities resulting in an improved salt-stress tolerance (Sofy et al., 2020). Similarly, in onions grown under salt stress, the application of GB resumed the restricted growth, water use efficiency, photosynthetic pigments, leaf gas exchange, and membrane stability index (Rady et al., 2018).

#### Polyamines

Polyamines (Pas) play a vital role in lessening the ill effects of abiotic stresses including salt stress. An increase of polyamines in plants on exposure to stress conditions is perceived to be beneficial (Antoniou et al., 2021). Priming using Pas is one of the desirable techniques to improve salt tolerance. Seeds of salt-sensitive and non-sensitive rice cultivars were primed with spermine (SPM) and spermidine (SPD) and seedling growth and transcriptome profiling were analyzed by Paul and Roychoudhury (2017). The results showed that seedlings germinated from SPM and SPD primed seeds showed increased expression of antioxidant and osmolyte biosynthetic genes as well as ABA biosynthesis gene and ABA-inducible transcription factor in roots and shoots compared to non-primed seedlings. Additionally, photosynthetic efficiency was also increased due to the priming with SPM and SPD. Taïbi et al. (2021) reported an altered content of polyamines in *Phaseolus vulgaris* L. under salt stress, and the tolerant cultivar accumulated a higher content of spermidine while both cultivars showed different gene expression levels encoding enzymes associated with spermine biosynthesis. Antoniou et al. (2021) also noted that salt stress resulted in an increase in spermine in a salt-tolerant cultivar of *Medicago truncatulato* Gaertn. compared the sensitive cultivars. An altered gene expression related to biosynthesis and catabolism of polyamines was noted in *M. truncatula* cultivated under salt stress. This depicts that under salt-stress conditions, the exogenous application with polyamines could be a strategic action to improve the negative impact of salt stress. Xiong et al. (2018) reported that the application of putrescine (Put) to salt-stressed tea (*Camellia sinensis* L.) plants improved their photosynthetic activity and decreased ROS production. This was depicted by a decreasing trend in SOD, POD, and CAT activities. The treatment with Put to salt-stressed cucumber plants improved photosynthetic capacity by regulating photochemical efficiency of PSII, hence mediated the injurious effects of salinity stress (Zhang et al., 2009). In salt-stressed lemon plant, the Put application decreased salt-stress-induced rise in MDA, proposing the involvement of Put in the protection of the plasma membrane (Khorshidi and Hamedi, 2014). In guava seedlings, the Put applications decreased CAT and POX activities and improved plant growth compared to the un-stressed, control plants (Ghalati et al., 2020). The exogenous spermidine application increased salt tolerance in Kentucky bluegrass, which was related to the increased the activity of antioxidant enzymes and decrease in MDA levels in salt-treated plants (Puyang et al., 2015). Exogenously applied spermidine to salt-stressed cucumber and ginseng plants improved the salinity tolerance by improving antioxidant enzymes and osmoprotectants (Duan et al., 2008; Parvin et al., 2014). The application of spermidine to salt-stressed chrysanthemum lowered the uptake of Na^+^ and enhanced the photosynthetic capacity, enzymatic ROS scavenging capacity, the osmotic and ionic balance, and cells’ membrane balance (Zhang N. et al., 2016). In salt-alkali stressed tomato plants, the exogenous spermidine application decreased MDA content and O_2_^⋅–^ generation rate and improved the components associated with the ascorbate-glutathione cycle (Zhang Z. et al., 2016). Sang et al. (2016) saw a boost in photosynthesis in salt-stressed cucumber following the applications of spermidine than that of non-treated salt-stressed plants. Qian et al. (2021) studied the impact of exogenous spermidine on the *Gladiolus gandavensis* grown under salt stress. The authors noted an improvement in photosynthetic pigments, gas exchange, antioxidant enzyme activity, and expression of antioxidants and osmoprotectants-related genes.

#### Proline

Proline is a natural osmoprotectant in plants having a vital role in the regulation of abiotic stresses. The application of Pro to salt-treated crop plants has been observed to benefit the plants. Wani et al. (2019) inspected the combined role of Pro and/or 24-epibrassinolide (EBL) on salt-stressed mustard plant and observed improved plant growth through increased activity of antioxidants along with the regulation of photosynthesis under salt stress. In eggplant, Shahbaz et al. (2013) evaluated the impact of the Pro application under salt stress. The authors observed an improved growth and water use efficiency in salt-stressed eggplant. In *B. juncea* L., the foliar application of Pro mitigated salt stress *via* increasing antioxidant capacity. While working with salt-stressed tomato, Kahlaoui et al. (2018) observed that the application of Pro at flowering stage improved salt-stress tolerance *via* enhancing biosynthesis of Pro, increasing soluble protein contents, activating glutamine synthetase, and decreasing proline oxidase. The combined application of Pro and SA on salt-stressed cucumber improved the salt tolerance by improving endogenous Pro and decreasing Na^+^ uptake in plants than untreated salt-stressed plants (Mugwanya et al., 2022).

#### Trehalose

Sugars, such as non-reducing disaccharide trehalose (Tre) are present in minute quantities in plants and possess vital importance in plant growth and productivity under common and stress conditions (Sarkar and Sadhukhan, 2022). It acts as an osmoprotectant under stress conditions including salt stress. Plants vary in their ability to accumulate Tre under salt-stress conditions. Hence, plant priming with Tre shows improved salt-stress tolerance. For instance, Samadi et al. (2019) tested the Tre application on strawberry. Upon treatment with Tre, the salt-stressed plants were able to maintain plant biomass, photosynthesis, and chlorophyll fluorescence traits, and reduced shoot Na^+^ accumulation, MDA and O_2_^⋅–^. Moreover, the Tre application triggered a boost in trehalase enzyme activity and endogenous Pro; phenolics and CAT, SOD, and GPX activities depicting the mitigating role of Tre in salt-stress persuaded oxidative stress. Feng et al. (2019) evaluated the Tre application on salt-stressed tomato plants and found that an improved salt-stress tolerance was associated with decreasing starch content and increasing soluble sugar content and abscisic acid (ABA), and genes related to their metabolism were induced, resulting in increased plant growth and biomass compared to non-treated salt-stress plants. Xie et al. (2022) observed that the application of Tre to tomato improved salt-stress tolerance by enhancing enzymatic activity related to Tre metabolic pathway. Furthermore, the Tre application counteracts the salt-stress induced oxidative stress in tomato plants *via* boosting antioxidant activities and related gene expression in salt-stressed tomato (Xie et al., 2022).

### Vitamins

#### Alpha-tocopherol

Tocopherols belonging to the vitamin E group are biosynthesized by plants and regulate plant abiotic stress tolerance as an antioxidant and signaling molecule (Ali et al., 2022). Naqve et al. (2021) evaluated the impact alpha-tocopherol on salt-stressed okra [*Abelmoschus esculentus* (L.) Moench] and observed an improvement in growth and yield *via* increasing the activity of antioxidants and via accumulation of Pro and GB. Moreover, the authors observed that alpha-tocopherol eased the hostile effects of salt stress by decreasing Na^+^ uptake, MDA, and H_2_O_2_ levels while boosting the uptake of K^+^ and Ca^2+^. El-Bassiouny and Sadak (2015) evaluated the impact of salt stress on flax (*Linum usitatissimum* L.) cultivars and observed a reduction in LP and activities of polyphenol oxidase, peroxidase and improved the SOD and CAT activities. In salt-stressed *P. vulgaris*, the exogenous application of alpha-tocopherol improved plant growth, physio–biochemical characteristics, mineral nutrients, soluble sugars and Pro, AsA, glutathione endogenous tocopherol, SOD, CAT, and guaiacol peroxidase (GPOX, E.C. 1.11.1.7) antioxidants than untreated controls (Hemida et al., 2017). In onion grown under salt stress, the application of alpha-tocopherol improved salt tolerance *via* upregulation of antioxidant activity (Semida et al., 2016). In faba bean, alpha-tocopherol applications improved salt-stress induced stunted growth *via* improved chlorophyll fluorescence, N, P, K, and Ca, yield, and leaf and stem anatomy (Semida et al., 2014).

#### Ascorbic acid

Seed priming with AsA has been shown to increase salt-stress tolerance in plants. Alves et al. (2021) observed that tomato seeds priming with AsA exhibited improved tolerance to salt stress, and such tolerance was associated with the modification of the antioxidant defense system and ROS scavenging enzymes. In salt-stressed sweet peppers, El-Beltagi et al. (2022) examined the impact of foliar applied AsA and observed an improved growth and fruit yield *via* enhancing the photosynthetic pigments, polyphenoloxidase (PPO, E.C. 1.10.3.1) activity, and APX activity. Azizi et al. (2021) evaluated the impact of AsA on *Calendula officinalis* L. and observed that AsA can ameliorate salt stress and increase the carotenoids, phenolic, and flavonoid concentrations in the flowers. Wang et al. (2019) studied the salt-stressed okra seedlings under the influence of the foliar application of AsA and noted that it improved growth indicators, chlorophyll, carotenoids, and activities of antioxidant enzymes while decreasing EL, H_2_O_2_, and LP. In a recent study, Chen et al. (2021) evaluated the AsA-induced photosynthetic ability of tomato seedlings and observed that application of AsA lessened the photoinhibition and reduced the damaging effects on photosynthesis by promoting chlorophyll synthesis and decreasing oxidative stress to the chloroplast by controlling redox state in chloroplast and disintegrating excitation energy in the PSII antennae.

#### Thiamine

Thiamine, or vitamin B1, is recognized as an enzymatic co-factor that is known to be involved in vital metabolic pathways responsible for cell energy supply in plants. More recently, the pivotal role of thiamine in mitigating abiotic stress has been unraveled through various studies. Moreover, seed priming or the exogenous application of thiamine to plants under salt stress resulted in enhanced intracellular thiamine contents as well as an improved tolerance (Kaya et al., 2015). Thiamine application on maize plants grown under salt-stress enhanced photosynthetic pigments, reduced membrane permeability, MDA and H_2_O_2_ levels, and changed activities of some key antioxidant enzymes under a saline regime (Kaya et al., 2015). Rapala-Kozik et al. (2012) reported that upregulation of genes connected with the biosynthesis of thiamine and thiamine diphosphate (TDP)-dependent enzymes occurred in response to osmotic and salt stress in Arabidopsis. Furthermore, the TDP, along with TDP-dependent enzymes (α-ketoglutarate dehydrogenase, pyruvate dehydrogenase, and transketolase) were upregulated simultaneously. All these enzymes are part of the main metabolic pathways responsible for the extreme environmental conditions in plants. Moreover, Rahman et al. (2017) demonstrated that the thiamine biosynthesis increased as a result of its biosynthesis genes upregulation under salinity stress. In another study, Wouyou et al. (2017) reported an improvement in the accumulation of thiamine and other vitamins in Amaranthus leaves under salt stress. Tuna et al. (2013) examined that thiamine-treated maize plants showed alleviation of salinity effects by enhancing the antioxidant activities. Furthermore, thiamine application minimized salt stress induced changes in protein metabolism of plants (El-Shintinawy and El-Shourbagy, 2001).

Spraying thiamine exogenously can enhance salt-stress tolerance in maize and sunflower (Hamada and Al-Hakimi, 2009). El-Metwally and Sadak (2019) reported an improved salt tolerance in faba bean plants (*Vicia faba* L.) which was found to be linked with enhanced Pro, free amino acids, and total carbohydrates. Furthermore, the authors observed an enhanced leaf area index, improved yield and its attributes, and the chemical composition of the yielded seeds (El-Metwally and Sadak, 2019). The foliar application of thiamine on maize cultivars induced tolerance against the oxidative stress condition induced by salinity (Kaya et al., 2015).

### Elements

#### Silicon

Silicon (Si) is one of the most abundant elements found in the universe. It is part of the group of metalloids which include arsenic, antimony, boron, germanium, and tellurium. Various studies have depicted the impact of Si in improving plants’ ability to better withstand abiotic stresses (Sahoo et al., 2019; Zhu et al., 2019; Hoffmann et al., 2020; Tripathi et al., 2020; Dhiman et al., 2021). For instance, Alves et al. (2020) found that the application of Si on lettuce seeds improved germination rate, CAT, GR, and SOD activities, and decreased lipid peroxidation (LP) and H_2_O_2_ content under salt stress. Guo et al. (2020) suggested Si application to mitigate the salt-induced reduction in seed germination and to lessen the salt-induced oxidative injury to seedlings of cucumber. In sweet basil plant, the application of Si improved salt tolerance *via* restoring osmotic balance and improving photosynthesis, redox homeostasis, osmolyte assimilation, and adjusting the expression of salt-stress-inducible proteins (Farouk et al., 2020). Zhu et al. (2020) studied the role of exogenous Si application in salt-treated cucumber. They reported an improved tolerance against salinity stress after Si treatments. Si application reduced oxidative stress *via* the regulation of Pro at different stress phases through δ 1-pyrroline-5-carboxylate synthetase (*P5CS*) and proline dehydrogenase (*ProDH*) activities and *P5CS* gene expression and interaction with cytokinin. Furthermore, Si application induced the expression of several cytokinin dehydrogenases (*Csa1G589070* and *Csa4G647490*) and isopentenyl transferase (*Csa3G150100* and *Csa7G392940*) genes which could be important for the regulation of cytokinin under salt stress (Zhu et al., 2020). Abdelaal et al. (2020) demonstrated that Si application improved plant water status, photosynthesis, and production of antioxidants that not only reduced oxidative stress but also improved fruit yield of *Capsicum annuum*. In salt-stressed tuberose (*Polianthes tuberosa*) plants, supplementation with Si enhanced growth and yield and induced the antioxidant defense system, while increasing postharvest vase life (Shahzad et al., 2021). In general, seeds priming with Si activates the antioxidant machinery, improves water status through accumulation of osmoprotectants, maintains the balance of nutrient elements, and enhances photosynthetic pigments and plant growth under salt stress (Abdel Latef and Tran, 2016).

From all these studies, seed priming with Si is a practical strategy for improving plant salt tolerance. As the second most abundant element on the earth, its application would not cause environmental problems. However, molecular aspects need to be explored for an improved understanding of mechanisms related to Si-mediated salt tolerance.

#### Nanoparticles

The field of nanotechnology has shown great potential for handling salt stress in crop plants. Khan et al. (2020) reported the mediating effect of priming with silver nanoparticles (AgNPs; 20 mM) in salt-stressed pearl millet. The application of carbon NPs to lettuce promoted seedling tolerance to 150-mM NaCl stress (Baz et al., 2020). Primed plants showed higher production of antioxidants, Pro, Na^+^/K^+^ and relative water content and minimum oxidative damage. Pérez-Labrada et al. (2019) tested copper (Cu) NPs based priming on tomato plants grown under salinity and reported improved performance *via* lowering Na^+^/K^+^ ratio, increased phenol, vitamin C, and GSH production, and increased phenylalanine ammonia lyase (PAL), APX, GPX, SOD, and CAT activities. Hojjat and Kamyab (2017) evaluated the potential of Ag NPs toward seed germination of fenugreek under salt stress. They observed that seeds treated with Ag NPs showed enhanced germination under salt-stressed conditions. In ajowan [*Trachyspermum ammi* (L.) Sprague ex Turrill], the foliar applications of nano-Fe_2_O_3_ (3 mM) and SA (1 mM) ameliorated the negative impacts of salinity by improving K^+^/Na^+^ ratio, endogenous SA production, and Fe content and by increasing SOD, CAT, POX, and osmolytes (Abdoli et al., 2020). Moradbeygi et al. (2020) observed that *Dracocephalum moldavica* L. plant supplemented with iron oxide (Fe_2_O_3_) based NPs showed an improved salt-stress status *via* improving enzymatic and non-enzymatic activities in roots and shoots and by decreasing ROS accumulation. Gohari et al. (2020a) reported salt-stress tolerance in *D. moldavica* L: seedlings after titanium dioxide NPs. The combined application of Pro and halloysite nanotube (Hal) mitigated the oxidative stress in sweet basil plants and improved their agronomic performance *via* enhancing their physiological potential and activities of antioxidants such as APX, SOD, and GP (Masoudniaragh et al., 2021). Cotton seeds primed with poly(acrylic acid)-coated cerium oxide nanoparticles (PNC) produced seedlings with decreased ROS accumulation and increased root length and fresh weight under salt stress (An et al., 2020). Transcriptome analysis showed that both ROS and Ca^2+^ mediated signaling could be implicated in nanoparticle-mediated salt tolerance as ROS enzymatic pathways (POD, GST, and PRC) as well as calcium transporter CAX1, and calcium binding EF-hand family genes were regulated. Additionally, Mg content in roots of cotton seedlings increased, terpene synthase genes were upregulated.

Based on the current knowledge, plant priming with NPs can be used as potential agents for salt-stress management in crop plants. However, from the perspective of sustainability, it is better to adopt the green-based NPS as plant priming agents and evaluate their role in mediating salt-stress tolerance.

### Polymers

#### Chitosan

Chitosan is a poly-(D)-glucosamine that is found in crustacean shells, insects, and fungi. Behind its use in biomedical areas, chitosan has been used in agriculture with beneficial effects. Application of chitosan has been reported to boost tolerance to salt stress. Jabeen and Ahmad (2013) evaluated the impact of safflower (*Carthamus tinctorius* L.) and sunflower (*Helianthus annuus* L.) seed treatment with chitosan under salt stress and reported enhanced tolerance *via* reduction of antioxidant activity at lower oxidative stress conditions in both plant species. In *Silybum marianum*, priming with chitosan NPs improved photosynthesis pigment, antioxidant enzymes, and osmoprotectant Pro under salt stress, hence imparting salt-stress tolerance (Mosavikia et al., 2020). Sheikhalipour et al. (2021) demonstrated that foliar treatment of Chitosan-Selenium NPs at 20 mg L^–1^ reduced the salt-induced alterations in Bitter Melon *via* boosting antioxidants, osmoprotectants, K^+^ uptake, and relative water content, and decreasing Na^+^, H_2_O_2_, and MDA. In salt-stressed tomato, Attia et al. (2021) evaluated the influence of chitosan mixed with different organic acids including AsA, acetic acid, citric acid, and malic acid. They observed increases in carotenoids, antioxidants, and osmoprotectants including soluble sugars, proteins and Pro, total phenols, and AsA and decreases in H_2_O_2_, MDA and Na^+,^ especially in response to AsA or citric acid-based chitosan application. Geng et al. (2020) evaluated the effects of chitosan on salt-stressed creeping bentgrass (*Agrostis stolonifera* L.). They reported improvement in relative water content, water use efficiency, photosynthesis, and photochemical efficiency. Furthermore, the decrease in salt-induced oxidative damage was mitigated by increased polyamines, antioxidants (SOD, POD, and CAT), and by altering sucrose accumulation and metabolism, total amino acids, γ-aminobutyric acid (GABA), and glutamic acid accumulation (Xu et al., 2021). Moreover, Na^+^ accumulation was decreased in leaves. Furthermore, chitosan application upregulated the expression of genes encoding Na^+^/H^+^ exchangers and boosted salt overly sensitive (SOS) pathways under salinity stress (Geng et al., 2020). In sweet pepper, Alkahtani et al. (2020) observed an improvement in the salt-stress induced a decline in chlorophyll *a* fluorescence (*F*_*v*_/*F*_*m*_ ratio), chlorophyll concentration, relative water content, enzyme activity, Pro, and fruit yield following the application of chitosan. Further application of chitosan and rhizobacteria decreased oxidative stress as indicated by MDA, EL, O_2_^⋅–^, and H_2_O_2_. Zhang et al. (2021) evaluated the impact of chitosan applications on salt-stressed cucumber and observed an improvement in growth *via* an increase in K^+^, photosynthetic pigments, Pro, and soluble sugar contents, enhanced peroxidase and CAT activities, and alleviated membrane LP, in comparison with salt-stressed untreated plants.

All these reports show the benefits of chitosan in salt-stress tolerance of plants. The available studies depict that the phenomenon may be based on the regulation of ions intake, antioxidant defense, and salt overly sensitive pathway. However, molecular aspects need to be explored to better understand the positive phenomenon of chitosan-mediated salt-stress tolerance in crop plants.

## Possible molecular basis of priming induced salt tolerance

This review highlights a total of 20 chemical priming agents enabling plant tolerance to salt stress. These agents have been categorized into groups: Plant growth regulators, reactive agents, osmoprotectants, vitamins, mineral elements, and polymers. Due to such a diversity of chemicals and also limited availability of molecular information on the action of some agents, it is difficult to speculate if there is a common molecular mechanism underlying different priming agent-mediated salt tolerance in plants. However, as discussed above, priming by different agents results in some common physiological and biochemical responses in plants and also induced a large number of gene expressions. The majority of the genes are defense-related ones, including enzymatic antioxidants, osmoprotectants, and polyamines. Such similarity may indicate that there may be a cross-talk network among the priming agent induced primed state, which requires further investigation.

It is generally agreed that salt-stress induced oxidative stress, i.e., an increase in the cellular levels of ROS which not only can adversely affect plant growth and crop yield but also cause programmed cell death. Elevated ROS can also trigger plant defense responses by upregulation of stress-protective genes and accumulation of metabolites and proteins to counteract the increased ROS and control the ROS at certain levels to sustain cell functions. The priming agents, such as plant hormones, reactive agents, and selected nanoparticles can act as a signal that modulates stress response and enhance plant ability to withstand salt stress. On the other hand, the roles of osmoprotectants include the induction of relevant gene expression to produce more osmoprotectants for reducing shoot Na^+^ accumulation and activation of enzymes to produce endogenous Pro, phenolics and CAT, glutathione peroxidase (GPX E.C. 1.11.1.9), and SOD to reduce salt-induced oxidative stress. As to the vitamins, they are either enzymatic co-factors or have antioxidant activities, thus, reducing salt-induced oxidative stress. Silicon improved salt tolerance by restoring osmotic balance and redox homeostasis and improving photosynthesis. Similarly, chitosan can mitigate salt stress by increasing antioxidant enzymes (SOD, POD, and CAT) and polyamines.

Seeds or plant priming is to use the priming agents to induce plants to enter the primed state before salt stress. As a result, primed plants are able to rapid activation of defense responses when challenged by salt stress, exhibiting increased tolerance to the stress. The primed state is reached molecularly by the activation of relevant TFs, epigenetic regulation, such as DNA methylation, chromatin remodeling, histone modification, and activation of transposable elements as well as post-translation regulation (Nair et al., 2022), which are outside of the scope of this review.

## Conclusion

**FIGURE 2:**
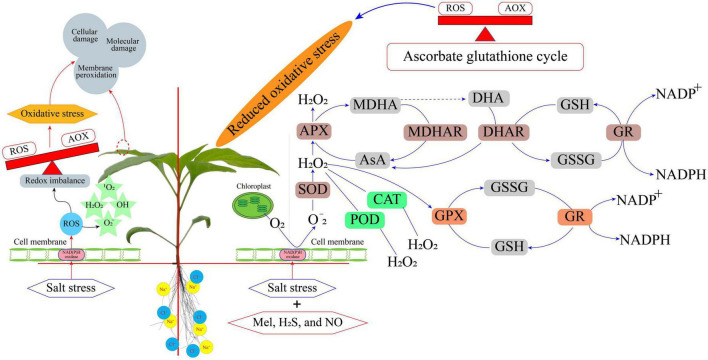
An overview of priming mediated mechanisms related to salt-stress tolerance.

Salt stress generally causes a reduction in plant growth and hence it is considered a barrier to achieving food security. Genetic approaches are powerful tools that can increase the tolerance of plants to salinity; thus, new techniques for genome-editing, such as the clustered regularly interspaced short palindromic repeat (CRISPR)-associated system (Cas), are very promising (Zhang et al., 2019; Yue et al., 2020). However, this implies large investments in research time and increased costs. Furthermore, genetically modified organisms (GMOs), including plants for human consumption, are not yet socially acknowledged in many countries. Likewise, the classical genetic strategies to find cultivars of species that are more resistant to salinity are also plausible approaches. However, this also takes a long time for analysis at the field level. Therefore, the use of priming could be a complementary and useful strategy at the agronomic level. The plant priming practice is demonstrating tremendous potential to mediate abiotic stresses in a wide range of crop plants. Plant priming with different molecules as discussed above has been shown to mediate salt stress tolerance. Moreover, the application of priming techniques is quick, efficient, low cost, and time-saving compared to the conventional strategies for managing salt stress (Figure 2). Most of the priming agents are environmentally friendly to a wide range of plant species, although some of them, such as NPs need to be critically evaluated in terms of their environmental safeness on a long-term basis. The priming agents not only can be used for seed treatment but also can be used only at some critical time before stress challenges. On the whole, adopting plant priming can fulfill the desire for reducing agrochemicals and assist researchers to refine the solutions for sustainable agriculture. Research on plant priming using novel compounds must expand toward the molecular level for a better understanding of the involved mechanisms induced under salt stress. Although this review presents the main molecules used for priming, other molecules appear as prospects in priming technology. Therefore, more investigations in plant–substrate systems are needed to decipher the role of plant priming at the molecular level and guide its application for improving crop tolerance to salt stress.

## Author contributions

FZ conceptualized and wrote the original draft. All authors reviewed and revised the manuscript.
